# Preparing Sections of Teeth for Histology and Bacteriology

**Published:** 1891-06

**Authors:** Vida A. Latham

**Affiliations:** Ann Arbor, Mich.


### Preparing Sections of Teeth for Histology and Bacteriology.

### PART I.--HISTOLOGY PRACTICAL.

VIDA A. LATHAM, ANN ARBOR, MICH.

Part of a paper with additions read at The Student's Dental Society U. of M.

The reason dental histology is as yet in its commencement lies, I believe, chiefly in the fact that the methods of preparation are very tedious, or at least appear so to the would-be investigator, and the methods used are not perhaps as good as they might be, had more time been given to this specialty. The course on histology is too brief to be of much use to the dental student, and very often the structure of the teeth is only *very* briefly noticed, if at all. Therefore it may be well if we can find some allusion to the many methods mentioned and a short notice of some of them be collected in this paper, where they can be of use for some future time. It would be well if authors of papers who write in this line of work, would give the practical methods at the same time, and thus enable one to have specimens to illustrate the articles and help in proving some of the ideas often advanced at the meetings, and in this way give the value of an entirely different opinion on the subject.

As regards re-agents and stains, the fewer and simpler that obtain a given result the better, though when the student is able to use the ordinary stains *well*, he can always try others. A special room for microscopical work should always be had, if possible, or a corner of a room with a good light where all this work can be done without any interruption. If possible, oil-cloth should cover the floor, which can easily be wiped over with a damp cloth, to take up the dust, for cleanliness is *absolutely essential.* A strong, firm table with a few drawers, is very convenient, and should be painted with a dull black paint to act as a background for the examination of specimens. The cost of apparatus will vary from fifty to one hundred dollars, including the microscope, incubators, sterilizers, glass, stains, &c.—though if the student is unhandy in making odd apparatus the cost will be considerably more. The best advice is, only to get a little apparatus to begin with, and add to it as the requirements become necessary.

Dental microscopy, for convenience is divided into:

(1.) Hard sections. (2.) Softened sections. (3.) Pulp sections. (4.) Development sections.

(1.) *Hard sections*: (*a*) The old method of cutting a fresh tooth into sections with a saw, grinding down fine, thoroughly cleansing, polishing, and mounting in Canada balsam, is one of the best methods for hard sections, when not required to show the pulp and more delicate structures.

(*b*.) Ground glass, using with it in the early stage, fine ground pumice stone, which is also especially convenient for grinding rough shells, like those of the lobster or crab is a good method. First soak the jaw of a mouse, rat, weasel, etc., in a solution of balsam in benzole; allow it to become hard, and then grind down as above. Very beautiful sections showing the teeth *in situ* may be made.

(*c*.) Another way is to take a tooth, the fresher the better, and if care is used, you can make two or three sections from it by using a new, thin, fine file to cut through the enamel, wet with turpentine and soft soap, and then use a broad-frame saw for cutting through the dentine. The section should be about one-eighth of an inch in thickness. Next flatten one side on a fine revolving corundum wheel (Ash's No. 9, fine) till it is ground quite flat. Then polish that side with the most perfect polish it is capable of receiving, on a piece of wet buff leather, with some putty-powder on it. Now take a piece of stout plate glass about two inches square, put a little old, and consequently tough, Canada balsam on it, warm, and spread it a little larger than your section. Let the balsam cool till it is "tacky;" then press the polished side of the tooth into close contact with the glass. When quite cold, proceed to grind as in the first part of the operation till you get the required thinness, when the side may be also polished. The hard balsam around the section supports and protects the edges, which will not be fractured (unless heated too much) and made jagged and untidy. In *not* putting the tooth on the glass till the balsam is somewhat cool, you prevent the polished surface from being covered by fine cracks, and also the balsam from running into the tubular structure of the dentine. A useful embedding mixture I find for grinding teeth and bone is made or composed of colophony resin, two parts, wax, one part; melt them together, and embed a piece of tooth or bone in the mixture, to a piece of glass. Then rub down thoroughly on sandstone, polish with emery paper of different degrees of fineness, then putty, on the glass.

(*d.*) Another method is to grind the sections till thin, holding it against the wheel with a smooth, firm cork, or it may be attached to the cork by aid of the resin mentioned above. It is recommended to endeavor to split the tooth with a pair of splitting-forceps in such a manner that the line of division passes through the centre of the cavity; place the thin section between two plates of ground glass with water and some levigated pumice powder, and by a rotary motion of the upper glass gradually rub the section down till it is thin enough to be examined with even high powers. Great care must be used when finishing, as an extra turn of one of the glasses may ruin the whole section. To finish with, old polished glass is the best, and when sufficiently thin mount in Canada balsam. Canada balsam is not, strictly speaking, soluble in alcohol, but is converted by it into a white condition, therefore the plate and thin section attached to it, is placed in alcohol, and in a few hours the section is easily detached without fracture, but is coated with the altered balsam, every particle of which must be removed with a clean camel-hair pencil, kept constantly wet with spirit; if this is not done, the specimen will be messy and muddled when mounted. When quite clean it may be placed in alcohol till the specimens are to be mounted. The reason why the section is not put in some complete solvent of balsam, is that, by so doing, we avoid the very thing we should have brought about, *i. e.*, to mount our section without the highly refractive balsam rendering it invisible, and that is why the alcohol is recommended. If the sections are desired to be transparent some solvent of balsam, oil of turpentine is good, till it becomes quite clear, if left under a bell glass. Then chloroform can be used and followed with not very thin balsam. To mount sections after embedding in the colophony resin, which if desired can be used even up to the proportion of ten parts of the resin to one of wax; and if the sections are damaged and not likely to hold together, the colophonium need not be dissolved, as when pure it is little inferior to Canada balsam. In this case the slide should be warmed very gently, or some drops of chloroform run over it before the cover-glass is put on.

*Method:* (1.) Saw a piece off a tooth or bone (held in a vice), rub it flat on a fine file; polish the flat surface on a fine hone, water-of-Ayr stone being preferable.

(2.) Fasten the section on to a piece of plate-glass, one inch square, with a cement made by melting six parts of button "lac" with one part Venice turpentine.

(3.) File the section down moderately thin, and then reduce further on the water-of-Ayr stone, examining under a low power from time to time.

(4.) Soak the section off with strong methylated spirit, or alcohol; wash *thoroughly* in clean spirit, and dry between tissue, which if desired may be placed between blotting paper.

(5.) Make a thin solution of white shellac in spirit, filter and keep in a stoppered bottle. Dip the section in this solution, drain, and lay on a cold plate under a bell-glass. In about half an hour it will be dry.

(6.) Mount in cold Canada balsam in benzole in preference to hard balsam, in order to avoid heating the section, as that would give it a tendency to curl; but as the melting point of shellac is higher than balsam, the latter may be used if thought desirable.

Gysi (Alfred) (I.) recommends a freshly-extracted tooth be ground down with a coarse corundum-wheel to a pretty thin lamella, according as a transverse or longitudinal section is desired; grind the section (one-side) evenly on a fine stone, polish on the same side on leather, coated with some fine polishing powder, till no rags are visible anywhere. Then cement the thin section polished side down on a glass slide, with some thick Canada balsam; heat very slightly, and firmly press the lamella against the glass, around the section cement some exceedingly thin pieces of covering glasses. It is convenient to cement on the other side of the glass a piece of burnt cork, by which to manage the slide during the grinding process. Now grind the section by hand on a fine and perfectly flat stone until all the thin covering glasses are evenly touched, ensuring a uniform thinness of section. Remove the cover glass pieces and grind the section still thinner, when thin enough it usually detaches itself. All this grinding must be done with water on a water-stone, then polish it on this newly ground side. The thin section is now washed in alcohol and every particle of polishing powder is brushed off with a fine camel's hair pencil, and again, wash in several changes of alcohol to ensure thorough cleanliness, and place in absolute alcohol for five minutes or more to remove every trace of water, then transfer to oil of cloves to clear it, and mount in Canada balsam. In my experience the clove oil renders the section liable to curl up, and to obviate this difficulty the sections may be placed, when clean, from the ordinary alcohol or even dilute, and place it in oil of cajeput or bergamot, though I prefer the former, this renders it unnecessary to pass the section through absolute alcohol or clearing with oil of cloves, as they may be mounted directly from the cajeput oil in ordinary balsam, dissolved in either benzole or chloroform. The Canada balsam may, if desired, after evaporation to glassy hardness in the ordinary way, be dissolved in oil of cajeput, in place of the usual solvents, as benzole, chloroform, xylol, etc. Gysi (I.) recommends Canada balsam, styrax or monobromide of naphthaline.

Preparations of enamel may be stained in the same manner as dentine.

*Mounting Teeth in Balsam.*--There are several ways of doing this: (1.) Plunge the section for a moment in white shellac and quickly withdraw it. The alcohol evaporates, leaving the porus structure completely *occluded* and protected from the balsam, however liquid it might be. (2.) Remove the section from the alcohol, let it dry protected from the dust, when nearly dry, soak well in distilled water to fill the tubules, lamallæ, and canaliculi with water; then dry its surfaces by wiping with a clean warm finger so that all moisture is taken from them, when the section may be mounted in rather firm balsam, without the structure being destroyed.

By either method specimens of abnormal dental histology may be satisfactorily preserved.

(3.) A simple way to mount any histological specimens is to place a slip or glass slide on a mounting card so as to center it, on the slip place a drop of Canada balsam, spread it evenly with a needle over the surface likely to be covered by the cover glass. On a lifter draw the section from the clearing agent, drain off the superfluous and place the edge of the lifter on the glass slide in such a way that when the section is drawn from it, it will lie over the center of the slide. Then take a clean cover glass with a pair of cover glass forceps and on its edge, put a drop of Canada balsam, invert the cover and place the balsamed edge against the glass slide at an angle of about forty five degrees and *very* gently lower the cover glass into place by drawing the forceps when almost parallel to the glass slide. Avoid pressing down with needles weights etc; as the cover will be brought to its natural place by the evaporation of the balsam solvent.

When dry, the slides are ready to ring with Hollis' Glue or some other varnish.

[TO BE CONTINUED.]

